# Extraction, Purification, Structural Characterization, and In Vitro Simulated Digestion of Polysaccharides from *Elaeagnus angustifolia*

**DOI:** 10.3390/foods15132318

**Published:** 2026-06-30

**Authors:** Hulalai Ayideng, Shihua Huang, Bibinuer Yaermaimaiti, Nuerxiayier Nazhaer, Naweire Yasen, Lina Zeng, Buweizuohere Tayier, Aiziguli Mulati

**Affiliations:** College of Food Science and Pharmacy, Xinjiang Agricultural University, Urumqi 830052, China; 13209012831@163.com (H.A.); 15310666887@163.com (S.H.); 15099561635@163.com (B.Y.); n17881217821@126.com (N.N.); 17799619206@163.com (N.Y.); 15739812520@163.com (L.Z.); 17799134855@163.com (B.T.)

**Keywords:** *Elaeagnus angustifolia* polysaccharide, polysaccharide, ultrasound-assisted extraction, separation and purification, structural characterization, in vitro simulated digestion

## Abstract

To exploit medicinal and edible plant resources, this study investigated the extraction, structural characterization, and in vitro digestion of *Elaeagnus angustifolia* polysaccharide (EAP). Despite the nutritional value of this polysaccharide, its digestive properties remain unclear. Ultrasound-assisted extraction was optimized via response surface methodology. Crude EAP was purified by AB-8 macroporous resin purification and decolorization, followed by deproteinization and dialysis. The purified product (91.07% total sugar; 2.37% protein) was characterized by ultraviolet (UV) spectroscopy, Fourier-transform infrared (FT-IR) spectroscopy, high-performance liquid chromatography (HPLC), and gel permeation chromatography (GPC), and its digestion profile was assessed using a three-stage in vitro model (INFOGEST 2.0). Under optimal conditions, the crude polysaccharide yield (based on ethanol-precipitated solid; 87.83% total sugar) reached 2.44 ± 0.01%. EAP was identified as a pyranose-type polysaccharide, with glucose, mannose, and galactose as the predominant monosaccharides (relative molar proportions, 0.470:0.199:0.081, normalized to the total detected monosaccharides), with a weight-average molecular weight of 1.739 × 10^5^ g·mol^−1^. In vitro digestion revealed negligible digestibility in the oral phase (0%), and low digestibility in the gastric (0.55%) and intestinal (2.76%) phases, with a cumulative digestibility of 3.30%. This marked resistance to gastrointestinal digestion indicates EAP is a partially digestible polysaccharide with potential prebiotic properties. The demonstrated resistance to gastrointestinal breakdown provides a theoretical basis for the high-value utilization of EAP in functional foods as a potential fermentable substrate for gut microbiota modulation.

## 1. Introduction

Polysaccharides are structurally diverse macromolecules composed of monosaccharide residues linked by glycosidic bonds, widely distributed in plants, fungi, algae, and bacteria. Metabolic syndrome has emerged as a defining global health challenge of the 21st century. A recent systematic review and Bayesian modeling analysis of 3236 data points encompassing 45.5 million adults revealed that global prevalence more than doubled between 2000 and 2023, rising from 11.9% to 28.4% overall [1]. By 2023, an estimated 1.54 billion adults were affected—846 million women (31.0% prevalence) and 692 million men (25.7% prevalence). The burden is disproportionately concentrated in East Asia and South Asia, while prevalence escalates steeply with age, urbanization, and national income level. Current pharmacological interventions remain limited by adverse effects and poor compliance, creating an urgent need for safe, microbiota-targeted dietary alternatives. In this context, edible fungal polysaccharides have attracted extensive attention for their functions in enhancing immunity, alleviating intestinal barrier damage, and relieving inflammation [2]. Notably, emerging research reveals that edible fungal polysaccharides ameliorate obesity, diabetes, and inflammatory bowel disease by modulating gut microbiota [2], offering a promising microbiota-targeted approach to address this escalating metabolic syndrome epidemic.

*Elaeagnus angustifolia* L. is widely distributed across Northwest China, subtropical Europe, parts of North America, and the Himalayas [3,4,5,6]. This species exhibits diverse pharmacological effects, including anti-inflammatory, antioxidant, anti-fatigue [7,8,9], insecticidal, anti-ulcer, and anti-mutagenic activities, as well as probiotic proliferation-enhancing, wound-healing, and cartilage-repairing properties [10,11]. Phytochemical studies have revealed that *Elaeagnus angustifolia* contains a rich array of bioactive constituents, including alkaloids, amino acids, ketones, vitamins, polyphenols, and polysaccharides [12,13,14,15]. Specifically, alkaloids such as elaeagnin (a tetrahydroharman-type *β*-carboline), N-methyl harmol, harman, and dihydroharman are mainly concentrated in the root, bark, and aerial parts [16]. Amino acids are abundant in the fruit pulp and peel, with glutamic acid (12.32–16.22 g/100 g protein), arginine (5.89–11.87 g/100 g protein), lysine (3.65–10.02 g/100 g protein), and aspartic acid (4.70–12.14 g/100 g protein) as predominant components [17]. Ketone compounds, such as 4,5,6-trihydroxyhex-3-en-2-one (61% relative abundance in purified fractions) and acetol, have been identified in fruit extracts [18]. Vitamins, including vitamin A, vitamin K, and B-complex vitamins, are present in flower methanol extracts, while ascorbic acid (vitamin C) reaches 5.6 mg% and β-carotene (provitamin A) 17.5 mg% in dried ripe fruits [16]. Polyphenols are highly concentrated in the leaf ethyl acetate fraction (290.97 mg GAE/g) and fruit peel (958.14 mg GAE/100 g), with chlorogenic acid (40.8–41.9 mg/g dry extract), rutin (75.77 mg/g), quercetin, and caffeic acid as major contributors [17,19]. Polysaccharides, represented by PEA-1 (MW 9113 Da, composed of rhamnose 37.1%, glucose 35.4%, galactose 19.6%, xylose 6.3%, mannose 1.6%) and PEA-2 (MW 5020 Da), exhibit potent antioxidant activity, with 2,2-diphenyl-1-picrylhydrazyl (DPPH) scavenging rates up to 88.1% [19,20]. Among these, *Elaeagnus angustifolia* polysaccharide (EAP) is considered a primary active component responsible for many of the abovementioned pharmacological effects. However, systematic studies on EAP extraction optimization, structural characterization, and digestive behavior are currently lacking, severely restricting its development as a functional food ingredient.

The extraction process is the prerequisite for studying polysaccharide structure and function. Traditional methods, including hot water, acid/base, and enzyme-assisted extraction, generally suffer from long processing time, low efficiency, high reagent consumption, and low yield [21], which are inadequate for large-scale production. Ultrasound-assisted extraction has gained prominence as a green, efficient alternative, where acoustic cavitation disrupts cell walls and accelerates mass transfer, achieving higher yields with reduced solvent and energy consumption [22]. To address these limitations, novel green extraction techniques such as supercritical fluid extraction, microwave-assisted extraction, and ultrasound-assisted extraction have been developed [23]. Different extraction processes significantly affect the yield, primary structure, and functional properties of polysaccharides [24,25]. For instance, systematic comparisons of water, alkaline, sequential, and enzymatic extractions for plant cell-wall polysaccharides have demonstrated that alkaline conditions disrupt hydrogen bonds to release high-molecular-weight fractions, enzymatic treatments cleave glycosidic bonds to enhance oligosaccharide yield, and sequential acid/alkaline treatment enriches specific pectic fractions [25]. Such extraction-dependent structural variations underscore the necessity of optimizing extraction methods to obtain polysaccharides with desired physicochemical properties and biological functions.

The intestine is the primary site for polysaccharide digestion and absorption, where digestive characteristics directly determine in vivo bioactivity. High-molecular-weight polysaccharides resist human digestive enzymes; however, upon reaching the colon, these non-digestible compounds serve as fermentable substrates for gut microbiota, thereby conferring health benefits to the host [26]. Therefore, numerous studies have expanded from molecular structure and bioactivity to gastrointestinal digestive behavior.

In vitro simulated digestion models can rapidly and accurately characterize polysaccharide behavioral changes in the human digestive environment, providing important evidence for evaluating nutritional value and application potential [26]. These models enable standardized, reproducible assessment of polysaccharide stability and fermentability, which is essential for predicting functional potential and guiding product formulation. However, systematic studies on the purification process, spatial structure, and in vitro digestion characteristics of EAP are currently lacking worldwide. This research gap severely restricts the in-depth development and industrial application of this distinctive resource.

We hypothesize that ultrasound-assisted extraction can optimize EAP yield while preserving its bioactive structure, and that EAP resists upper gastrointestinal digestion but undergoes colonic fermentation, supporting its potential as a microbiota-modulating agent for metabolic syndrome.

This study establishes a complete research system of “extraction–purification–structure–digestion” for large-fruit oleaster polysaccharide, filling the relevant research gap. The results can provide technical parameters for large-scale preparation, clarify digestive patterns in the gastrointestinal environment, and offer a scientific basis for functional food formulation design.

## 2. Materials and Methods

### 2.1. Materials and Reagents

*Elaeagnus angustifolia* polysaccharide (EAP) used in this study was extracted and purified from *Elaeagnus angustifolia* samples collected from Southern Xinjiang, China. The prepared EAP was freeze-dried, crushed, and sieved through a 100-mesh screen for subsequent experiments. Petroleum ether (60–90 °C), anhydrous ethanol, phenol, concentrated sulfuric acid, trichloroacetic acid, and n-butanol were of analytical grade and purchased from Sinopharm Chemical Reagent Co., Ltd. (Beijing, China). Coomassie brilliant blue G-250, bovine serum albumin, glucose, AB-8 macroporous resin, DEAE-52 cellulose, Sephadex G-100, *α*-amylase, pepsin, pancreatin, bile salts, and DNS reagent were obtained from Shanghai Yuanye Bio-Technology Co., Ltd. (Shanghai, China). Potassium bromide (KBr, spectroscopic grade) was purchased from Sinopharm Chemical Reagent Co., Ltd. (Beijing, China). Ultrapure water was used throughout.

### 2.2. Experimental Methods

#### 2.2.1. Raw Material Pretreatment

The dried fruits of *Elaeagnus angustifolia* L. were washed, pitted, dried, crushed, and sieved to obtain fruit powder. A 100 g aliquot of the powder with constant weight was extracted twice with 500 mL petroleum ether (60–90 °C) under reflux for 2 h each time to remove lipids. After filtration, the residue was dried at 50 °C and stored for further use.

#### 2.2.2. Ultrasound-Assisted Hot Water Extraction of Crude Polysaccharides

First, 1.0 g of pretreated *Elaeagnus angustifolia* fruit powder was weighed. The powder was prepared by grinding dried fruit in a grinder and passing through a 100-mesh sieve. Single-factor experiments were conducted to investigate the effects of ultrasonic power (100, 120, 140, 160, and 180 W), liquid-to-solid ratio (20:1, 30:1, 40:1, 50:1, and 60:1 mL/g), ultrasonic time (10, 20, 30, 40, and 50 min), and ultrasonic temperature (30, 40, 50, 60, and 70 °C) on the polysaccharide extraction yield. The optimal conditions from single-factor experiments were an ultrasonic power of 140 W, liquid-to-solid ratio of 40:1 mL/g, ultrasonic time of 30 min, and ultrasonic temperature of 50 °C. The optimal level from each single-factor experiment was selected as the center point for subsequent response surface design. Based on the single-factor experimental results, a four-factor, three-level response surface experiment was designed using the Box–Behnken design (29 runs, including 5 center points) with the factor levels of ultrasonic power (120, 140, and 160 W), liquid-to-solid ratio (30:1, 40:1, and 50:1 mL/g), ultrasonic time (20, 30, and 40 min), and ultrasonic temperature (40, 50, and 60 °C) to optimize the extraction process parameters, with the polysaccharide extraction yield as the response value. The Box–Behnken design was selected over Taguchi design because it enables fitting of quadratic models to capture curvature and factor interactions, avoids extreme factor combinations that might cause polysaccharide degradation, and provides uniform prediction variance across the experimental domain. The extraction solution was filtered, concentrated to one-fifth of its original volume by Rotary evaporator (Yarong Biochemical Instrument Factory, Shanghai, China, RE-5205A), mixed with four volumes of anhydrous ethanol, and allowed to stand overnight at 4 °C. The mixture was then centrifuged at 6000 rpm for 10 min, and the precipitate was collected and freeze-dried using a vacuum freeze dryer (Ningbo Scientz Biotechnology Co., Ltd., Ningbo, China, SCIENTZ-10N/C) to obtain crude polysaccharides. The polysaccharide extraction yield was calculated according to the following formula:

(1) $$\mathrm{yield}\;(\%)\;=\;\frac{\mathrm{Mass}\;\mathrm{of}\;\mathrm{crude}\;\mathrm{polysaccharide}}{\mathrm{Mass}\;\mathrm{of}\;Elaeagnus\;angustifolia\;\mathrm{sample}}\times 100\%$$

### 2.3. Separation and Purification of Crude Polysaccharides

#### 2.3.1. Deproteinization Treatment

The crude polysaccharides were dissolved in distilled water to prepare a 10 mg/mL solution. Three deproteinization methods were applied, with three replicates per group: trichloroacetic acid method (mixing with an equal volume of 20% trichloroacetic acid), n-butanol method (mixing with an equal volume of n-butanol), and n-butanol-trichloroacetic acid method (mixing with an equal volume of a 1:1 mixture of n-butanol and 20% trichloroacetic acid) [27]. Each mixture was shaken for 1 h, allowed to stand for 3 h, and centrifuged (6000 rpm, 10 min) to remove the precipitate. The protein content in the supernatant was determined using the Coomassie brilliant blue method [28], with bovine serum albumin (BSA) as the standard. Protein concentration was expressed as mg/mL, and the protein removal rate was calculated according to the following formula to select the optimal deproteinization method:

(2) $$\mathrm{Protein}\;\mathrm{removal}\;\mathrm{rate}\;(\%)\;=\;\frac{C_{1}\;-C_{2}}{C_{1}}\times 100\%$$

where C_1_ is the protein concentration before deproteinization (mg/mL), and C_2_ is the protein concentration after deproteinization (mg/mL).

#### 2.3.2. Decolorization Treatment

Purification via AB-8 macroporous resin adsorption was performed as described [29]. The resin was swollen in anhydrous ethanol for 24 h and washed with distilled water until the ethanol odor was eliminated; and then it was wet-packed into a column (2.6 × 40 cm, column volume ~200 mL). The deproteinized polysaccharide was freeze-dried and re-dissolved in distilled water to prepare a 10 mg/mL solution, which was loaded at 1.0 mL/min using a peristaltic pump (YZ1515x pump head, Shenchen, Baoding, China) with 1.6 mm wall thickness tubing. After loading, the column was eluted with distilled water at 1.5 mL/min, controlled by the same pump. The flow rates were digitally set and verified by volumetric calibration. Fractions were collected every 10 min, with a total run time of approximately 450 min. The fractions were concentrated by rotary evaporation and freeze-dried for subsequent analysis.

#### 2.3.3. Column Chromatography Purification

DEAE-52 cellulose was pretreated by alternating immersion in 0.5 mol/L NaOH and 0.5 mol/L HCl, washed with distilled water to neutrality, and wet-packed into a column (2.6 × 50 cm) before being equilibrated with distilled water [30]. The decolorized polysaccharides were prepared as a 4.0 mg/mL sample solution. After loading 5 mL of the sample, the column was eluted sequentially with distilled water and a 0.1–0.6 mol/L NaCl gradient [31] at a flow rate of 1 mL/min, with fractions collected every 8 min (8 mL/tube). The total run time was approximately 1500 min. The eluate was monitored at 490 nm using the phenol-sulfuric acid method [32] to plot the elution curve, and the major polysaccharide fractions were pooled. The pooled fractions were concentrated and loaded onto a Sephadex G-100 gel column (1.6 × 60 cm), which was eluted with distilled water at a flow rate of 0.8 mL/min, with fractions collected every 10 min (total run time: approximately 300 min) and monitored using the phenol-sulfuric acid method. Fractions showing a single symmetric peak were pooled, dialyzed (molecular weight cutoff of 3500 Da) for 48 h, and freeze-dried to obtain the EAP.

### 2.4. Physicochemical Properties of the Polysaccharide

#### 2.4.1. Total Sugar Content Determination

The total sugar content was determined using the phenol-sulfuric acid colorimetric method. A glucose standard solution (0.1 mg/mL) was prepared. Aliquots of 0, 0.2, 0.4, 0.6, 0.8, and 1.0 mL were pipetted into test tubes, and the volume was adjusted to 1.0 mL with distilled water. Then, 1.0 mL of 5% phenol solution and 5.0 mL of concentrated sulfuric acid were added rapidly. After mixing, the solution was allowed to stand at room temperature for 20 min, and the absorbance was measured at 490 nm using a UV-Vis spectrophotometer (Beijing Persee General Instrument Co., Ltd., Beijing, China, T700B) to plot the standard curve. The total sugar content of the sample was expressed as mg glucose equivalent per g dry weight (mg/g DW) based on the standard curve.

#### 2.4.2. Protein Content Determination

The protein content was determined using the Coomassie brilliant blue G-250 method. A bovine serum albumin standard solution (0.1 mg/mL) was prepared. Aliquots of 0, 0.2, 0.4, 0.6, 0.8, and 1.0 mL were pipetted into test tubes, and the volume was adjusted to 1.0 mL with distilled water. Then, 5.0 mL of Coomassie brilliant blue G-250 [28] solution was added. After mixing, the solution was allowed to stand at room temperature for 5 min, and the absorbance was measured at 595 nm using a UV-Vis spectrophotometer (Beijing Persee General Instrument Co., Ltd., Beijing, China, T700B) to plot the standard curve. The protein content of the sample was expressed as mg bovine serum albumin equivalent per g dry weight (mg/g DW) based on the standard curve.

#### 2.4.3. Reducing Sugar Content Determination

The reducing sugar content was determined using the DNS colorimetric method. A glucose standard solution (1.0 mg/mL) was prepared. Aliquots of 0, 0.2, 0.4, 0.6, 0.8, and 1.0 mL were pipetted into test tubes, and the volume was adjusted to 1.0 mL with distilled water. Then, 2.0 mL of DNS [33] reagent was added, and the mixture was heated in a boiling water bath for 5 min. After cooling, the volume was adjusted to 10 mL with distilled water, and the absorbance was measured at 540 nm using a UV-Vis spectrophotometer (Beijing Persee General Instrument Co., Ltd., Beijing, China, T700B) to plot the standard curve. The reducing sugar content of the sample was expressed as mg glucose equivalent per g dry weight (mg/g DW) based on the standard curve.

#### 2.4.4. Ash Content Determination

Crucibles (with lids) were placed in a muffle furnace, heated to 550 °C for 1 h, cooled to 200 °C, and then placed in a desiccator for 30 min before weighing. This process was repeated until constant weight was achieved (difference ≤ 0.5 mg between two consecutive weighings), and this weight was recorded as M0. Approximately 2 g of purified polysaccharide sample was weighed into the crucible, and the total weight was recorded as M1. The sample was carbonized until no white smoke was emitted and then placed in the muffle furnace and heated to 550 °C for ashing until constant weight was achieved. The final weight was recorded as M2. The ash content was determined by dry ashing according to AOAC Official Method 942.05 [29] and calculated according to the following formula:

(3) $$\mathrm{Ash}\;\mathrm{content}\;(\%)\;=\;\frac{\left(M2\;-\;M0\right)}{\left(M1\;-\;M0\right)\;}\times 100\%$$

### 2.5. Structural Characterization of the Polysaccharide

#### 2.5.1. UV Spectroscopy

The EAP was dissolved in ultrapure water to prepare a 0.5 mg/mL solution. UV spectra were recorded in the range of 200–900 nm, using ultrapure water as a blank to detect the presence of characteristic absorption peaks of nucleic acids or proteins.

#### 2.5.2. FT-IR Spectroscopy

A total of 2 mg of dried EAP sample was thoroughly ground with 200 mg of dried KBr powder in an agate mortar. The mixture was pressed into a pellet, and the FT-IR spectrum was recorded using a Fourier-transform infrared spectrometer (Bruker, Ettlingen, Germany, INVENIO-S) in the range of 4000–400 cm^−1^, with a resolution of 4 cm^−1^ and 32 scans. KBr was used as the background to identify characteristic functional groups and glycosidic bond types.

#### 2.5.3. Scanning Electron Microscopy

A small amount of polysaccharide powder was evenly adhered to a conductive carbon tape on an aluminum sample stage. The sample was sputter-coated with gold in a vacuum sputtering coater to improve conductivity. The surface morphology was observed using a scanning electron microscope (Zeiss Sigma, Carl Zeiss AG, Oberkochen, Germany) at an accelerating voltage of 5.00 kV, with a working distance of 4.0 mm. Micrographs were collected at magnifications of 500× and 2000× using the InLens detector. Energy-dispersive spectrometry (EDS) was performed using an Ultim Max detector (Oxford Instruments, Abingdon, UK) coupled with the SEM.

#### 2.5.4. Monosaccharide Composition Analysis

The monosaccharide composition [34,35,36,37] was determined by high-performance anion-exchange chromatography (HPAEC, Dionex ICS-5000+, Thermo Fisher Scientific, Waltham, MA, USA) with electrochemical detection. In brief, the sample (5 mg) was hydrolyzed in 3 M trifluoroacetic acid at 120 °C for 3 h. The hydrolyzed products were dissolved in 5 mL of ultrapure water with vortex mixing, and 50 μL of the solution was diluted with 950 μL of ultrapure water. The resulting 1 mL solution was centrifuged at 12,000 rpm for 5 min (5430R, Eppendorf, Hamburg, Germany), and the supernatant was filtered through a 0.22 μm Millipore membrane. The filtrate was then analyzed by HPAEC-ED equipped with a Dionex Carbopac PA20 column (150 × 3.0 mm) (Thermo Fisher Scientific, Waltham, MA, USA). The mobile phase elution was carried out at a flow rate of 0.3 mL/min using the following mobile phases: A, ultrapure water; B, 15 mM NaOH; C, 15 mM NaOH & 100 mM NaOAc. The injection volume was 25 μL and the column temperature was 30 °C. The elution gradient was set as follows: 0 min, A/B/C (98.8:1.2:0, *v*/*v*); 20 min, A/B/C (50:50:0, *v*/*v*); 30 min, A/B/C (50:50:0, *v*/*v*); 30.1 min, A/B/C (0:0:100, *v*/*v*); 46 min, A/B/C (0:0:100, *v*/*v*); 46.1 min, A/B/C (0:100:0, *v*/*v*); 50 min, A/B/C (0:100:0, *v*/*v*); 50.1 min, A/B/C (98.8:1.2:0, *v*/*v*); 80 min, A/B/C (98.8:1.2:0, *v*/*v*).

The molecular weight and molecular weight distribution of the EAP were determined using high-performance size-exclusion chromatography coupled with multi-angle laser light scattering and refractive index detection (HPSEC-MALLS-RI). The mobile phase was 0.1 mol/L NaNO_3_ containing 0.02% NaN_3_ at a flow rate of 0.5 mL/min. The column temperature was 40 °C, and the injection volume was 100 μL (sample concentration of 2 mg/mL). The system was equipped with a light scattering detector (NEON, wavelength 658.1 nm, fused silica cell) and a differential refractive index (dRI) detector. Water was used as the solvent with temperature correction enabled. Data acquisition and analysis were performed using ASTRA 7.3.0 software, and the dn/dc value was set to 0.138 mL/g [34,38].

### 2.6. In Vitro Simulated Digestion Experiment

#### 2.6.1. Preparation of Simulated Digestive Fluids

Simulated salivary fluid, simulated gastric fluid, and simulated intestinal fluid were prepared the standardized INFOGEST 2.0 in vitro digestion method [39]. The specific formulas were as follows:

(1) Simulated salivary fluid (SSF): KCl 1.491 g/L, KH_2_PO_4_ 0.636 g/L, NaHCO_3_ 1.428 g/L, MgCl_2_·6H_2_O 0.066 g/L, (NH_4_)_2_CO_3_ 0.048 g/L, adjusted to pH 7.0 with 1 mol/L HCl. *α*-Amylase was added before use to a final concentration of 75 U/mL.
(2) Simulated gastric fluid (SGF): KCl 0.515 g/L, KH_2_PO_4_ 0.122 g/L, NaHCO_3_ 2.100 g/L, NaCl 2.752 g/L, MgCl_2_·6H_2_O 0.049 g/L, (NH_4_)_2_CO_3_ 0.048 g/L, CaCl_2_·2H_2_O 0.022 g/L, adjusted to pH 3.0 with 1 mol/L HCl. Pepsin was added before use to a final concentration of 2000 U/mL.
(3) Simulated intestinal fluid (SIF): KCl 0.507 g/L, KH_2_PO_4_ 0.108 g/L, NaHCO_3_ 5.714 g/L, NaCl 2.244 g/L, MgCl_2_·6H_2_O 0.066 g/L, adjusted to pH 7.0 with 1 mol/L NaOH. Pancreatin (to achieve a final trypsin activity of 100 U/mL) and bile salts (final concentration of 10 mmol/L) were added before use.

#### 2.6.2. In Vitro Digestion Experimental Design

The EAP was dissolved in distilled water to prepare a 2 mg/mL solution. Three groups were set up with three replicates each: Group A (experimental), 2 mL SSF + 2 mL polysaccharide solution; Group B (saliva blank), 2 mL SSF + 2 mL distilled water; and Group C (polysaccharide blank), 2 mL polysaccharide solution + 2 mL distilled water.

(1) Oral digestion phase: The tubes were incubated in a shaker at 37 °C and 120 rpm for 5 min. The enzymes were immediately inactivated by heating in a boiling water bath for 5 min after incubation.
(2) Gastric digestion phase: After oral digestion, the pH of the three groups (A, B, and C) was adjusted to 3.0 with 1 mol/L HCl. The tubes were then incubated in a shaker at 37 °C and 120 rpm for 120 min. The enzymes were immediately inactivated by heating in a boiling water bath for 5 min after incubation.
(3) Intestinal digestion phase: After 120 min of gastric digestion, the pH of the three groups was adjusted to 7.0 with 1 mol/L NaHCO_3_, and an equal volume of SIF was added. The tubes were incubated in a shaker at 37 °C and 120 rpm. Samples were collected at 30, 60, and 120 min (three replicates per group per time point), and the enzymes were immediately inactivated by heating in a boiling water bath for 5 min.

#### 2.6.3. Index Determination and Digestion Rate Calculation

The reducing sugar content in the samples at each stage was determined using the DNS method, and the total sugar content was determined using the phenol-sulfuric acid method. The polysaccharide digestion rate was calculated according to the following formula:

(4) $$\frac{\mathrm{Initial}\;\mathrm{total}\;\mathrm{sugar}\;\mathrm{content}\;-\;\mathrm{Residual}\;\mathrm{total}\;\mathrm{sugar}\;\mathrm{content}\;\mathrm{after}\;\mathrm{digestion}}{\mathrm{Initial}\;\mathrm{total}\;\mathrm{sugar}\;\mathrm{content}}\times 100\%$$

Statistical analysis was performed using one-way ANOVA. Normality of residuals was confirmed using the Shapiro–Wilk test (*p* > 0.05), and homogeneity of variance was confirmed using Levene’s test (*p* > 0.05) prior to ANOVA.

#### 2.6.4. Data Processing

Response surface experiment design and model analysis were performed using Design-Expert software (Version 13, Stat-Ease, Inc., Minneapolis, MN, USA). One-way analysis of variance (ANOVA) and multiple comparisons (Duncan’s test) were performed using SPSS 26.0 software. Data plotting and fitting were conducted using Origin 2021 software. The experimental results are expressed as the mean ± standard deviation (mean ± SD), and *p* < 0.05 was considered statistically significant.

## 3. Results and Analysis

### 3.1. Effects of Different Extraction Parameters on the Extraction Yield of EAP

#### 3.1.1. Single-Factor Effects of Ultrasonic Time, Liquid-to-Solid Ratio, Ultrasonic Power, and Ultrasonic Temperature on the Extraction Yield

**Figure 1:**
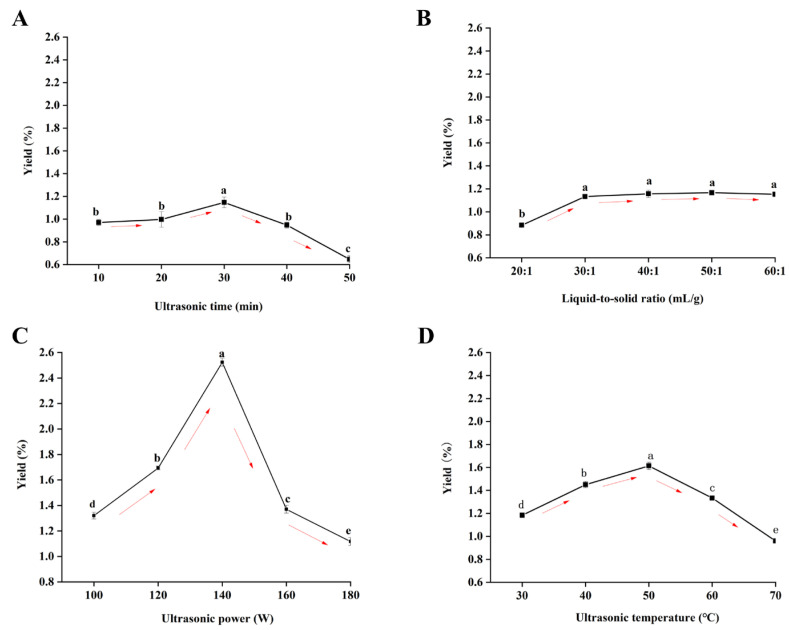
Effects of ultrasonic time (**A**), liquid-to-solid ratio (**B**), ultrasonic power (**C**), and ultrasonic temperature (**D**) on the extraction yield of EAP. Different superscript letters in the same panel indicate significant differences (*p* < 0.05).

Single-factor experiments showed that ultrasonic time, liquid-to-solid ratio, ultrasonic power, and ultrasonic temperature significantly affected the extraction yield of *Elaeagnus angustifolia* polysaccharides. Within an extraction time of 10–50 min, the yield increased with time, with an optimum at 30 min; excessive ultrasound damaged polysaccharide integrity, reducing yield. Increasing the liquid-to-solid ratio from 20:1 to 50:1 mL/g significantly increased the yield, which peaked at 50:1 mL/g; further increase to 60:1 mL/g resulted in a stable or slightly decreased yield, indicating that an excessively high ratio does not further improve extraction efficiency. Within the range of 100–180 W, the yield first increased and then decreased with increasing power, peaking at 140 W. Appropriate power disrupts cell walls via cavitation, promoting polysaccharide dissolution, whereas excessive power generates free radicals that cleave polysaccharide chains, reducing yield. As the ultrasonic temperature increased from 30 °C to 50 °C, the yield gradually rose and reached a maximum at 50 °C; further temperature increase led to structural degradation, oxidation, or decomposition, decreasing the yield (Figure 1).

#### 3.1.2. Response Surface Optimization of the Interactive Effects of Various Factors on the Extraction Yield

A quadratic polynomial regression model of the polysaccharide extraction yield (Y) with respect to four factors—ultrasonic power (A), ultrasonic temperature (B), ultrasonic time (C), and liquid-to-solid ratio (D)—was obtained by multiple regression fitting using Design-Expert software (Version 13, Stat-Ease, Inc., Minneapolis, MN, USA):

(5) $$Y=2.41+0.165A-0.158B-0.312C-0.138D-0.036AB+0.049AC+0.056AD-0.226BC+0.244BD-0.065CD-1.081A^{2}-1.102B^{2}-0.743C^{2}-0.915D^{2}$$

**Figure 2:**
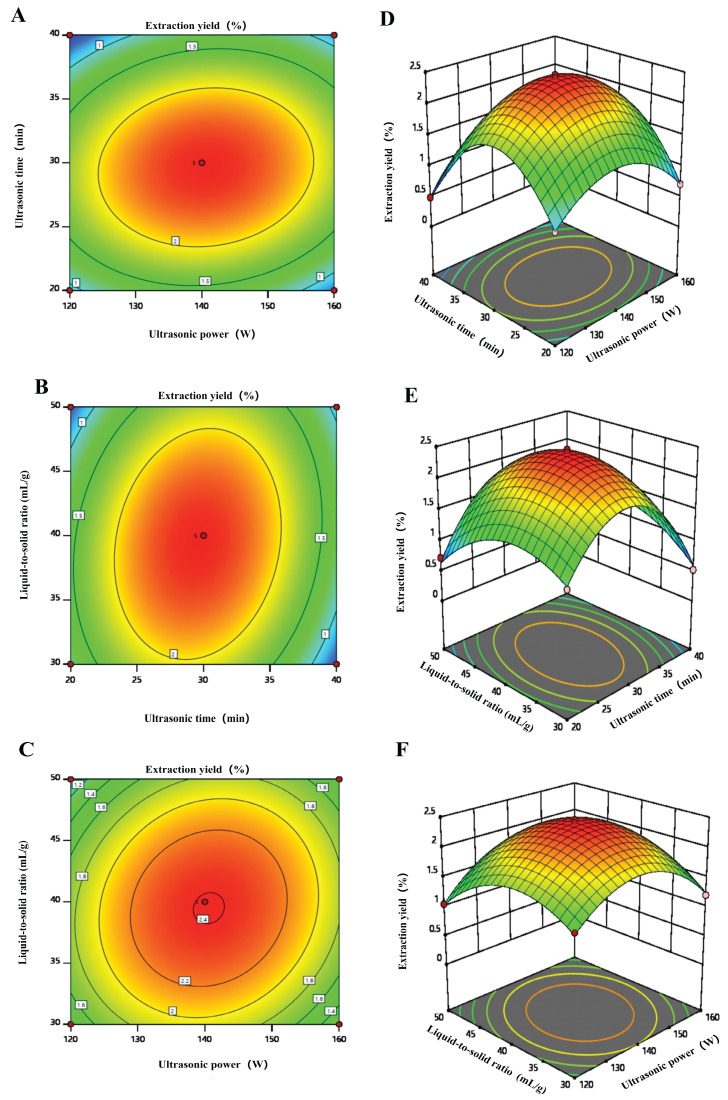
Response surface plots showing the effects of extraction parameters on polysaccharide extraction yield (ultrasonic temperature fixed at 50 °C). (**A**) Contour plot of ultrasonic power and ultrasonic time. (**B**) Contour plot of ultrasonic time and liquid-to-solid ratio. (**C**) Contour plot of ultrasonic power and liquid-to-solid ratio. (**D**) 3D response surface plot of ultrasonic power and ultrasonic time. (**E**) 3D response surface plot of ultrasonic time and liquid-to-solid ratio. (**F**) 3D response surface plot of ultrasonic power and liquid-to-solid ratio.

The Box–Behnken design matrix and experimental results are presented in Table 1. The analysis of variance (ANOVA) results for the regression model are shown in Table 2. The model F-value was 215.63 (*p* < 0.0001), indicating that the regression model was highly significant. The lack-of-fit F-value was 4.72 (*p* = 0.0741 > 0.05), which was not significant, indicating that the model fit the data well and could be used for the analysis and prediction of the EAP extraction process. The order of influence of the four factors on the extraction yield was as follows: ultrasonic time (C) > ultrasonic temperature (B) > liquid-to-solid ratio (D) > ultrasonic power (A). Among the interaction terms, AC, AD, BC, and CD were highly significant (*p* < 0.01), while AB and BD were significant (*p* < 0.05). Response surface plots for AC, AD, and CD are presented in Figure 2, with ultrasonic temperature fixed at its center point level (50 °C).

According to the regression model, the optimal extraction conditions were as follows: ultrasonic power of 140 W (A), ultrasonic temperature of 50 °C (B), ultrasonic time of 30 min (C), and liquid-to-solid ratio of 40:1 mL/g (D). Under these conditions, three validation experiments were conducted, yielding polysaccharide extraction yields of 2.432%, 2.447%, and 2.453%. The average extraction yield was 2.444 ± 0.0108%, indicating that this process is stable and reliable.

### 3.2. Effects of Different Purification Methods on the Separation Efficiency of EAP

#### 3.2.1. Effect of Different Deproteinization Methods on the Protein Removal Rate

The crude polysaccharide had an initial protein concentration of 3.909 mg/mL. Among the three deproteinization methods tested, the trichloroacetic acid method achieved the highest protein removal rate (96.84%), which was significantly superior to the other two methods. Therefore, this method was selected as the optimal deproteinization protocol (Table 3).

#### 3.2.2. Effect of AB-8 Macroporous Resin Decolorization on the Polysaccharide Retention Rate

**Figure 3:**
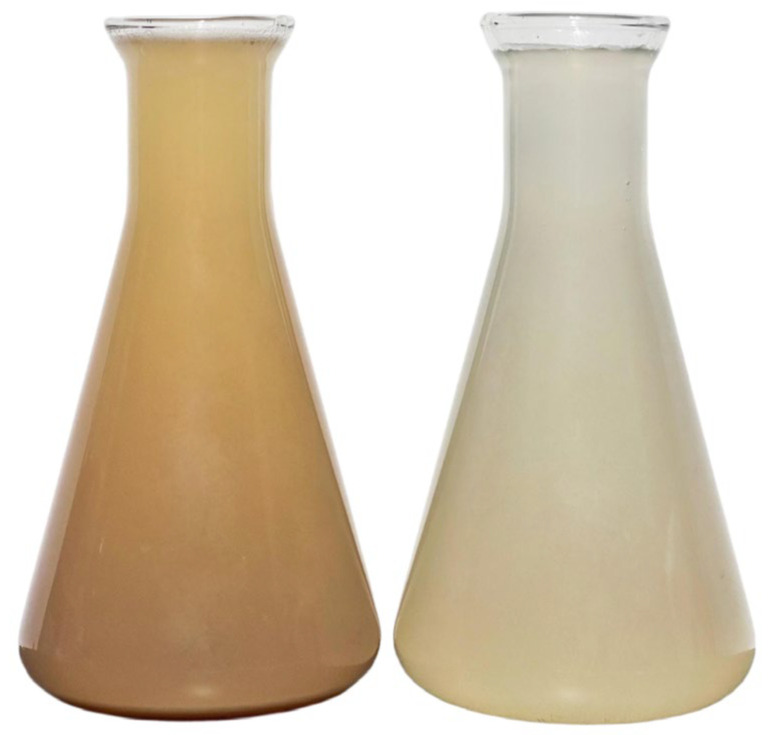
Decolorization of the crude polysaccharide extract by AB-8 macroporous resin ((**left**) before and (**right**) after).

AB-8 macroporous resin exhibited a remarkable adsorption effect on the pigments in the big fruit EAP. After decolorization, the polysaccharide solution became clear and transparent, with a polysaccharide retention rate of 76.04%, effectively reducing pigment interference with subsequent purification and structural characterization (Figure 3).

#### 3.2.3. Effect of Column Chromatography Purification on the Separation of Polysaccharide Components

**Figure 4:**
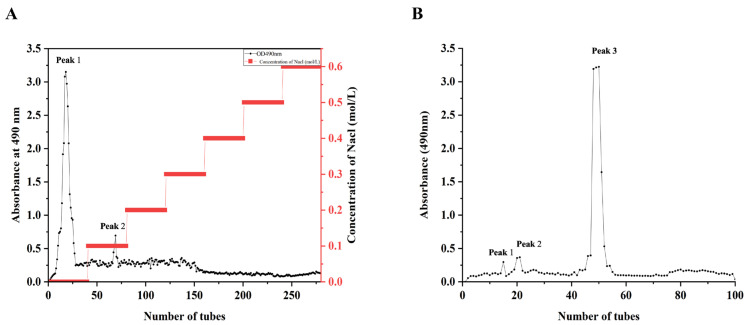
(**A**) DEAE-52 elution profile (H_2_O → 0–0.6 M NaCl step gradient). (**B**) Sephadex G-100 gel filtration profile.

After decolorization, the polysaccharides were subjected to DEAE-52 ion-exchange column chromatography with gradient elution using NaCl solutions of different concentrations. A major polysaccharide absorption peak (peak 1) appeared in the distilled water elution fraction, representing neutral polysaccharides, while a smaller absorption peak (peak 2) appeared in the 0.1 mol/L NaCl elution fraction, representing acidic polysaccharides (Figure 4A). The peak 1 eluate was collected, concentrated, and further purified using a Sephadex G-100 gel column. The elution curve showed a single symmetric peak (Figure 4B), indicating relatively homogeneous molecular weight distribution of the polysaccharide component. The fractions containing this peak were pooled, dialyzed, and freeze-dried to obtain a flocculent white EAP.

### 3.3. Effect of Purification on the Physicochemical Properties of EAP

The main physicochemical properties of EAP before and after purification are shown in Table 4. Note that total sugar includes both reducing and non-reducing sugars; therefore, the sum of individual components exceeds 100%. After purification, the total sugar content increased significantly from 87.83% to 91.07% (*p* < 0.01), while the protein content decreased extremely significantly from 39.93% to 2.37% (*p* < 0.01). Meanwhile, the reducing sugar content decreased significantly from 47.73% to 28.73% (*p* < 0.05), and the ash content decreased extremely significantly from 12.62% to 5.04% (*p* < 0.001). Notably, the standard deviations of the purified sample were markedly reduced compared to the crude sample, indicating that the purification process significantly improved the homogeneity and purity of the polysaccharide.

### 3.4. Structural Characterization of EAP

#### 3.4.1. UV Spectral Characterization of Polysaccharide Purity

**Figure 5:**
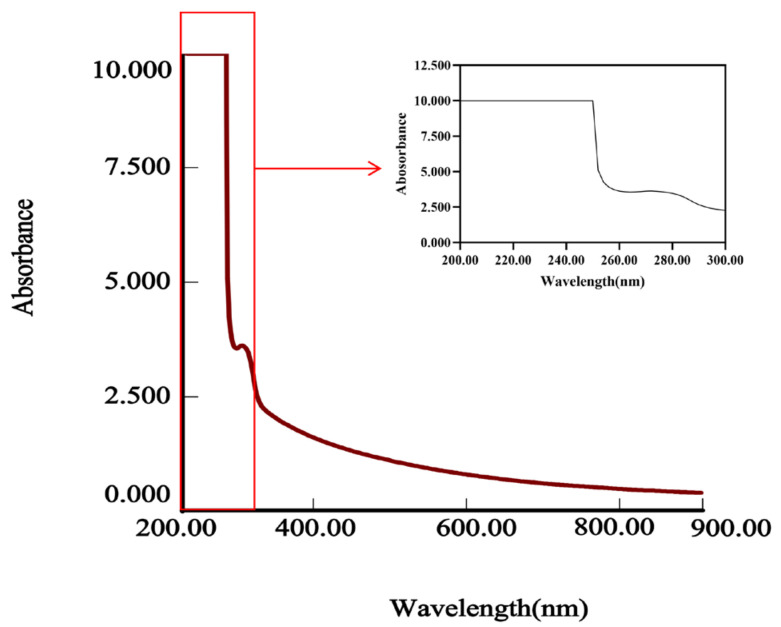
UV absorption spectrum of EAP. Strong absorption at ~200 nm indicates polysaccharide characteristics; absence of peaks at 260 and 280 nm confirms high purity.

The UV (Figure 5) absorption spectrum of EAP in the 200–400 nm range showed a strong terminal absorption at approximately 200 nm, which is characteristic of polysaccharides. No significant absorption peaks were observed at 260 nm or 280 nm, indicating that nucleic acid and protein impurities were largely removed and that the sample purity was high.

#### 3.4.2. FT-IR Characterization of Polysaccharide Functional Groups

**Figure 6:**
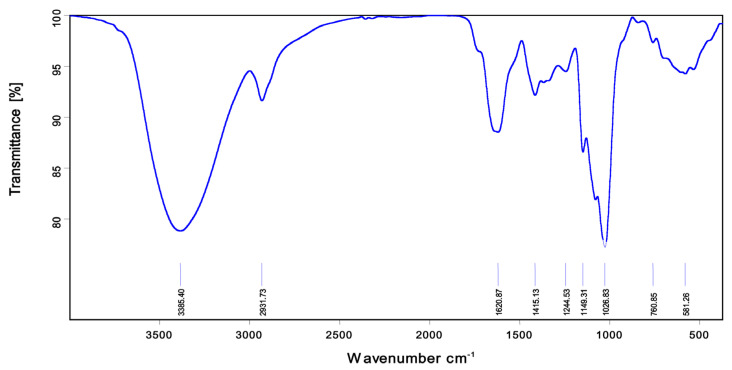
FT-IR spectrum of purified EAP. The absorption band at ~1040 cm^−1^ indicates pyranose-type polysaccharides; bands at ~1600 and ~1410 cm^−1^ indicate uronic acids in salt form (–COO^−^); and the absence of a peak at ~1730 cm^−1^ confirms no free carboxyl groups.

The infrared spectrum of polysaccharide is shown in Figure 6. According to previous studies, the broad and intense absorption band near 3385 cm^−1^ is attributed to the O–H stretching vibration, and the weak band near 2931 cm^−1^ is assigned to the C–H stretching vibration [40]. The strong absorption bands in the region of 1200–1000 cm^−1^ (observed at 1244, 1148, and 1026 cm^−1^ in this sample) are characteristic of a pyranose ring, indicating that the polysaccharide is a pyranose-type polysaccharide. No characteristic absorption peak was observed near 1730 cm^−1^, indicating the absence of free carboxyl groups of uronic acid. However, absorption bands were observed at 1620 cm^−1^ and 1415 cm^−1^, which may be attributed to the asymmetric and symmetric stretching vibrations of carboxylate groups (–COO^−^), suggesting the presence of uronic acids in salt form. The absorption band at 1620 cm^−1^ can also be attributed to bound water or a carbonyl group. Furthermore, the band at 1415 cm^−1^, assigned to the C–H bending vibration, is similar to the band near 1367–1370 cm^−1^ reported elsewhere, which was attributed to the C–H deformation vibration of CH_2_ groups [41]. The absorption bands at 780 cm^−1^ and 581 cm^−1^ are assigned to C–H out-of-plane bending vibrations or sugar-ring skeletal vibrations. No obvious absorption was observed near 850 cm^−1^ or 890 cm^−1^; therefore, the α/β anomeric configuration of the glycosidic bonds cannot be determined by FT-IR spectroscopy.

#### 3.4.3. SEM Characterization of Polysaccharide Micromorphology

**Figure 7:**
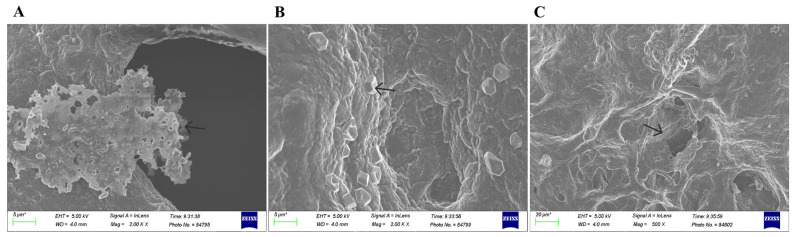
SEM images of purified EAP. (**A**,**B**) 2000×; (**C**) 500×. Scale bars = 5 μm (software-calibrated). Photo numbers are automatic file identifiers.

SEM images of the purified EAP are shown in Figure 7: (Figure 7A) at 2000× magnification, showing a porous, flaky, and folded interlaced loose network (arrow indicates flaky pore edge); (Figure 7B) another area at 2000× magnification, showing crystalline particles (arrow) embedded in the porous network; and (Figure 7C) at 500× magnification, showing a loose network structure with overlapping layers forming continuous porous channels (arrow indicates macropore), indicating well-developed pore structures and high porosity.

#### 3.4.4. Monosaccharide Composition Analysis for Polysaccharide Structural Type Determination

**Figure 8:**
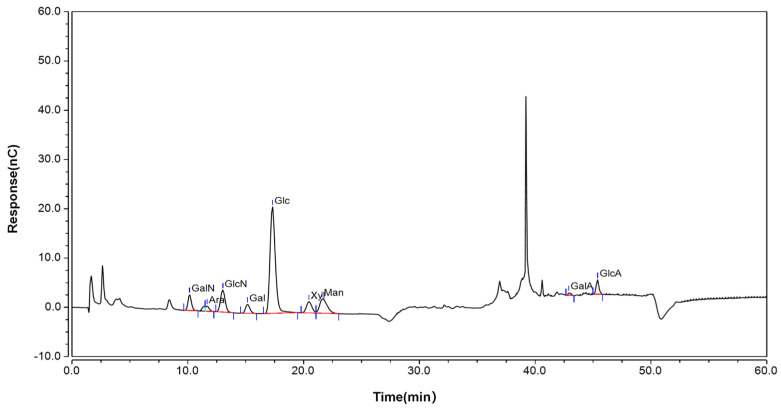
High-performance anion exchange chromatogram of EAP. Peaks: GalN = galactosamine, GlcN = glucosamine, Ara = arabinose, Gal = galactose, Glc = glucose, Xyl = xylose, Man = mannose, GalA = galacturonic acid, and GlcA = glucuronic acid. Fuc, Rha, Fru, and Rib were not detected. Blue vertical lines indicate the retention times of monosaccharide standards; the red line represents the fitted baseline.

The monosaccharide composition of EAP was determined by ion chromatography. The polysaccharide was mainly composed of glucose (Glu), mannose (Man), and galactose (Gal), with molar ratios of 0.470, 0.199, and 0.081, respectively (Figure 8). Small amounts of xylose (Xyl), glucosamine (GlcN), glucuronic acid (GluA), arabinose (Ara), galactosamine (GalN), and galacturonic acid (GalA) were also detected. Fucose, rhamnose, fructose, and ribose were not detected. These results indicated that EAP is a heteropolysaccharide primarily composed of neutral sugars, with glucose being the predominant monosaccharide (Table 5).

#### 3.4.5. Molecular Weight Determination for Evaluation of Polysaccharide Distribution Homogeneity

**Figure 9:**
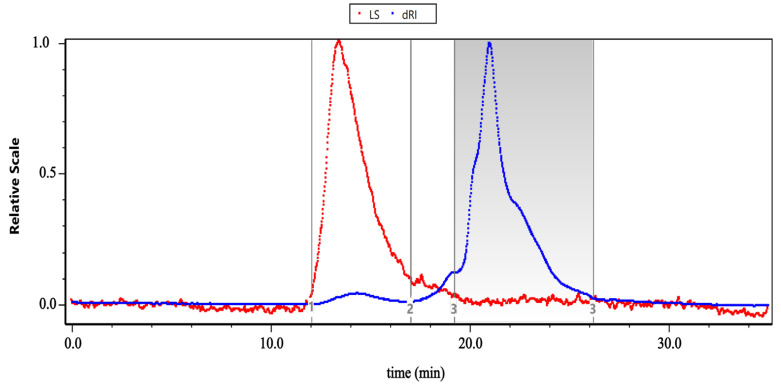
Molecular weight distribution of EAP determined by HPSEC-MALLS-RI. The gray shaded area indicates the integration range for Peak 3, the major polysaccharide component (90.8% mass fraction, Mw = 1.739 × 10^5^ g/mol). LS (light scattering, red) and dRI (differential refractive index, blue) signals are normalized to respective maxima.

The molecular weight (Figure 9) and molecular weight distribution of EAP were determined by HPSEC-MALLS-RI. Three chromatographic peaks were observed in the retention time range of 12.074–26.218 min. The main peak (peak 3) had a retention time of 19.241–26.218 min and accounted for 90.8% of the total mass, representing the major polysaccharide component. The mass fractions of peaks 1 and 2 were 4.1% and 5.1%, respectively. The molecular weight parameters of each peak are shown in Table 6. For the main peak (peak 3), the weight-average molecular weight (Mw) was 1.739 × 10^5^ g/mol, the number-average molecular weight (Mn) was 1.129 × 10^5^ g/mol, the peak molecular weight (Mp) was 5.975 × 10^5^ g/mol, and the polydispersity index (Mw/Mn) was 1.540, indicating a relatively narrow molecular weight distribution and high purity of the polysaccharide sample.

### 3.5. Effects of In Vitro Simulated Digestion on the Degradation Characteristics of EAP

The in vitro digestion experiment was conducted sequentially using simulated salivary fluid (SSF), simulated gastric fluid (SGF), and simulated intestinal fluid (SIF) to simulate the oral, gastric, and intestinal phases, respectively.

Note that “0 min” represents the initial sample before digestion (polysaccharide solution mixed with the corresponding digestive fluid and immediately sampled for enzyme inactivation).

#### 3.5.1. Effect of Different Digestion Stages on Total Sugar Content

**Figure 10:**
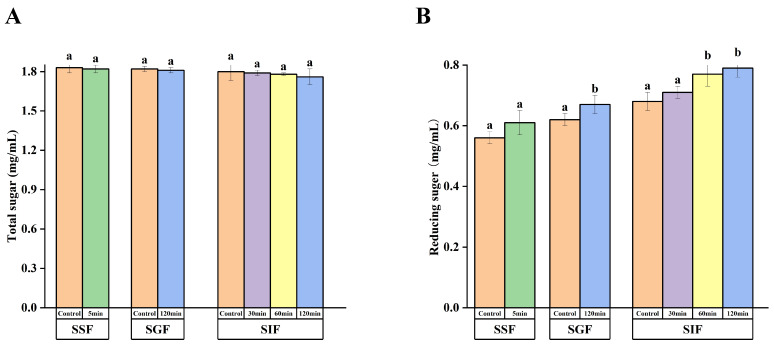
Changes in (**A**) total sugar content and (**B**) reducing sugar content of EAP during in vitro simulated digestion. SSF, simulated salivary fluid; SGF, simulated gastric fluid; SIF, simulated intestinal fluid. Different superscript letters in the same panel indicate significant differences (*p* < 0.05).

Analysis of the changes in total sugar content (Figure 10A) during in vitro simulated digestion revealed that after 5 min of salivary digestion, the total sugar content was 1.82 mg/mL. Although total sugar appeared relatively stable throughout digestion, this reflects minor absolute changes (1.82 to 1.76 mg/mL, ~3.3%) rather than complete resistance to degradation. After 120 min of gastric digestion, the total sugar content decreased slightly to 1.81 mg/mL (*p* > 0.05), indicating no significant degradation under gastric conditions. During the intestinal digestion phase, the total sugar content showed a continuous downward trend with increasing digestion time: 1.79 mg/mL at 30 min, 1.78 mg/mL at 60 min, and 1.76 mg/mL at 120 min (*p* < 0.05). These results indicated that the intestinal enzyme system has a certain degradation effect on EAP, but the degree of degradation is limited, and most of the polysaccharides can maintain their structural integrity and reach the colon.

#### 3.5.2. Effect of Different Digestion Stages on Reducing Sugar Release

Analysis of the changes in reducing sugar content (Figure 10B) during in vitro simulated digestion showed that after incubation of the polysaccharide solution with simulated saliva for 5 min, the reducing sugar content was 0.61 mg/mL, which was not significantly different from the initial value before incubation (0 min, 0.56 mg/mL) (*p* > 0.05). This finding indicates that salivary *α*-amylase had no obvious degradation effect on the EAP, and the polysaccharide remained structurally stable in the oral environment. After 120 min of gastric digestion, the reducing sugar content increased to 0.67 mg/mL, which was significantly higher than the value at the end of the salivary digestion stage (0.61 mg/mL) (*p* < 0.05), indicating that partial degradation of the EAP occurred under the synergistic action of the gastric acid environment and pepsin, with the cleavage of glycosidic bonds releasing small amounts of reducing sugars. During the intestinal digestion phase, the reducing sugar content continued to rise. Compared with the initial intestinal value (0 min, 0.68 mg/mL), the level at 30 min (0.71 mg/mL) was not significantly different (*p* > 0.05), while the levels at 60 min (0.77 mg/mL) and 120 min (0.79 mg/mL) were significantly higher (*p* < 0.05). The intestinal fluid contains various digestive enzymes, such as pancreatin, which can more effectively act on the EAP, promoting further hydrolysis of the polysaccharide chains and the continuous release of reducing sugars. Overall, the reducing sugar content showed a monotonically increasing trend throughout the entire digestion process, increasing from 0.56 mg/mL before salivary digestion to 0.79 mg/mL at the end of intestinal digestion. This confirmed that EAP can be gradually degraded in the gastrointestinal tract, with the intestinal enzyme system having the most significant degradation effect.

#### 3.5.3. Degradation Characteristics and Apparent Digestibility of EAP During In Vitro Digestion

**Figure 11:**
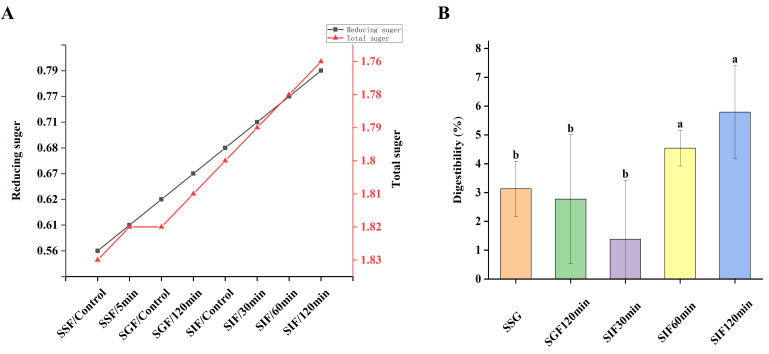
Changes in total sugar and reducing sugar contents of EAP during in vitro simulated digestion. (**A**) Dynamic changes in total sugar and reducing sugar. (**B**) Digestibility at different digestion stages (oral, gastric, and intestinal phases). SSF, simulated salivary fluid; SGF, simulated gastric fluid; SIF, simulated intestinal fluid. Different superscript letters in the same panel indicate significant differences (*p* < 0.05).

Based on the changes in total sugar content during in vitro simulated digestion, the apparent digestibility of EAP at each digestion stage was calculated. Using the total sugar content at the end of the oral phase (1.82 mg/mL) as the baseline, no significant total sugar consumption occurred in the oral phase, resulting in an apparent digestibility of 0%. Apparent digestibility was calculated relative to the respective phase baseline: gastric digestibility, 0.55% (1.82 → 1.81 mg/mL); intestinal digestibility, 2.76% (1.81 → 1.76 mg/mL); and cumulative gastrointestinal digestibility, 3.30% (1.82 → 1.76 mg/mL) (Figure 11B).

However, calculations based solely on total sugar consumption may underestimate the actual extent of glycosidic bond cleavage. The correlation analysis shown in Figure 11A reveals that the reducing sugar content increased from 0.56 mg/mL to 0.61 mg/mL (an increase of approximately 9%) during the oral phase. The minor increase in reducing sugars during the oral phase likely reflects the release of pre-existing low-molecular-weight oligosaccharides or free reducing sugars loosely associated with the crude polysaccharide preparation, rather than enzymatic hydrolysis of the polymer backbone. This interpretation is supported by three observations: (i) total sugar content remained unchanged (1.82 mg/mL), indicating no depolymerization of the main chain; (ii) EAP lacks the α-1,4-glucan structure required for salivary *α*-amylase specificity; and (iii) the reducing sugar increase (~9%) was quantitatively minor and did not progress with extended incubation. Thus, EAP exhibits oral-phase stability, a prerequisite for its potential as a colonic-fermentable substrate.

During the gastric phase, under the synergistic action of gastric acid and pepsin, the reducing sugar content further increased to 0.67 mg/mL, indicating limited polysaccharide degradation. Upon entering the intestinal phase, pancreatic enzymes and other enzyme systems played a dominant role, with reducing sugar content continuously rising to 0.79 mg/mL, while total sugar content decreased from 1.80 mg/mL to 1.76 mg/mL, demonstrating further hydrolysis of the main polysaccharide chain.

Integrating the data from both figures, the EAP can be classified as a “partially digestible polysaccharide”: the polysaccharide remains stable in the oral phase, slight degradation takes place in the gastric phase, and the most significant degradation occurs in the intestinal phase. However, with a cumulative total sugar consumption rate of only 3.30% throughout the entire gastrointestinal tract, the majority of the polysaccharide structure remains intact and reaches the colon, endowing it with the structural basis to exert prebiotic effects as a dietary fiber.

## 4. Discussion

### 4.1. Extraction and Purification of EAP

Ultrasound power of 100–180 W: Yield peaked at 140 W due to cavitation-promoted dissolution vs. free radical-induced chain cleavage at excessive power [42], a phenomenon similarly observed in pumpkin polysaccharide extraction, where optimal ultrasound conditions enhanced yield while excessive power caused structural degradation [43]. Temperature of 30–50 °C: Yield peaked at 50 °C; higher temperatures caused structural degradation [44,45], attributed to the destruction and degradation of polysaccharide structure under prolonged high-temperature conditions [46]. Time of 10–50 min: Results were optimum at 30 min; prolonged ultrasound impaired integrity through cumulative thermal and mechanical effects that compromise glycosidic bond stability [46,47]. Liquid-to-solid ratio of 20:1–60:1 mL/g: The plateau was at 50:1 mL/g, consistent with the principle that polysaccharide yield increases with solvent volume until reaching maximum dissolution rate, beyond which no further enhancement occurs [47].

Given parameter interdependence in complex hydrolysis environments [25], Box–Behnken design combined with response surface methodology was employed. This systematic optimization approach contrasts with conventional single-factor experiments and unoptimized protocols reported for other plant polysaccharides, such as the various extraction methods for cranberry pomace polysaccharides where alkaline extraction achieved higher yield (31.2%) but caused significant pectin defragmentation and debranching [25]. Optimal conditions were power at 140 W, time at 30 min, ratio at 40:1 mL/g, and yielding at 2.444%, consistent with model prediction. The moderate yield reflects the mild extraction conditions that preserve structural integrity, a trade-off similarly noted in optimized ultrasound extraction of *Anemarrhenae asphodeloides* polysaccharides, where BBD-RSM yielded 8.56% with preserved bioactivity [48].

Crude EAP was sequentially purified to obtain the final polysaccharide product (91.07% total sugar; 2.37% protein) used for structural characterization. Trichloroacetic acid (TCA) deproteinization (96.84%) significantly exceeded n-butanol (83.70%) and combined methods (87.29%) (*p* < 0.01) due to acidic glycopeptide bond cleavage. This efficiency surpasses the Sevag deproteinization commonly employed for polysaccharides such as RAP, where method comparison was not reported [48]. AB-8 resin purification and decolorization retained 76.04% polysaccharide while removing phenolics and small-molecule extractives. DEAE-52 elution confirmed that the polysaccharide is primarily neutral. The neutral nature of EAP differs from the acidic heteropolysaccharides (containing GalA and GluA) prevalent in many plant sources, including A. asphodeloides and cranberry pomace, suggesting distinct structural and functional properties. This purified product was employed in all subsequent structural and digestive characterization experiments.

### 4.2. Structural Characteristics of EAP

The polysaccharide is pyranose-type with glucose, mannose, and galactose dominance (0.470:0.199:0.081), differing from typical oleaster polysaccharides (arabinose/galactose-rich). This monosaccharide profile is simpler than the 10-component heteropolysaccharides reported for RAP (Man, Rha, Rib, GlcA, GalA, Glc, Gal, Ara, Xyl, and Fuc), reflecting both species-specific biosynthesis and the climatic stress (aridity and intense sunlight) of the Xinjiang cultivation region. This discrepancy reflects Xinjiang’s climatic stress (aridity and intense sunlight) and genetic characteristics.

FT-IR showed no 1730 cm^−1^ peak but exhibited 1620/1415 cm^−1^ carboxylate (–COO^−^) bands, indicating that uronic acids existed as salts, not free acids-resolving the apparent FT-IR vs. monosaccharide composition discrepancy. The absence of free uronic acids distinguishes EAP from pectin-rich polysaccharides such as those from cranberry pomace, where GalA predominates and contributes to acidic functionality [25].

High reducing sugar content (28.73%, MW 1.739 × 10^5^ g/mol) reflects (1) a highly branched structure with numerous reducing termini; (2) broad PDI (1.540), indicating low-MW oligosaccharide fragments; and (3) mild processing-induced degradation. The extensive structural characterization presented here (MW, PDI, branching pattern, and reducing termini) exceeds the monosaccharide composition-limited analysis reported for RAP, where detailed linkage patterns and molecular weight distributions were not investigated [48]. This results from intrinsic branching and extrinsic process factors.

### 4.3. In Vitro Simulated Digestion

Total sugar significantly decreased during digestion (*p* < 0.05), with reducing sugar increase indicating depolymerization/glycosidic bond cleavage [25]. The “oral stability—gastric partial degradation—intestinal continuous degradation” pattern relates to gastric acid hydrolysis and specific glycosidic linkages. Cumulative digestibility: 3.30%. This partial digestibility profile is novel for *Elaeagnus angustifolia* polysaccharides, as prior studies on related species focused primarily on extraction and composition without evaluating gastrointestinal fate.

SEM revealed a loose, porous morphology facilitating enzyme contact. Its classification was a partially digestible polysaccharide (between resistant *β*-glucan and digestible starch), attributable to neutral nature, specific monosaccharide composition, and glycosidic linkage types. The neutral charge and Glc-Man-Gal composition likely contribute to this intermediate digestibility, contrasting with acidic pectins that resist gastric digestion due to charge-mediated structural protection.

### 4.4. Limitations and Future Perspectives

Limitations: (1) This study focused on upper gastrointestinal digestion only; fecal fermentation simulation was not performed, which limits direct validation of the hypothesized prebiotic potential. (2) The ash content (5.04%) exceeds pharmaceutical-grade standards (<3%). The elevated ash content likely reflects the salt-form uronic acids identified by FT-IR (1620 cm^−1^ and 1415 cm^−1^ carboxylate bands), indicating that further optimization of desalting protocols is needed for pharmaceutical applications.

Future directions: (1) Fecal fermentation and animal studies are needed to validate prebiotic function and elucidate structure-activity relationships; (2) methylation analysis and 2D NMR for glycosidic linkage elucidation are needed, as applied in advanced pectin structural studies; (3) optimization of desalting procedures necessary to reduce ash content; and (4) development of EAP-based functional food formulations. The establishment of structure–digestibility relationships in this study provides a foundation for comparative investigations with other partially digestible polysaccharides and their metabolic fates.

### 4.5. Conclusion and Functional Food Implications

This study establishes a comprehensive database for the development of EAP as a functional food ingredient. The BBD-RSM optimized extraction protocol provides a scalable, parameter-controlled manufacturing process that preserves structural integrity—addressing the yield-integrity trade-off observed in harsh alkaline extraction of plant polysaccharides. The neutral charge and Glc-Man-Gal composition of EAP confer partial digestibility (3.30%), positioning it between resistant *β*-glucan and digestible starch for slow-release energy or targeted colonic delivery applications. The high reducing sugar content (28.73%) and porous morphology offer antioxidant capacity and encapsulation potential, respectively. These characteristics complement the existing literature on acidic heteropolysaccharides by introducing a distinct structural type with unique gastrointestinal behavior. Future fecal fermentation studies and animal trials, following the research trajectory suggested for related polysaccharides, will validate prebiotic functionality and enable health-claim applications.

## 5. Conclusions

This study systematically established a complete research framework for *Elaeagnus angustifolia* polysaccharides (EAP), encompassing extraction, purification, structural characterization, and in vitro digestive properties. A stable and reliable ultrasound-assisted extraction technology was successfully constructed, yielding 2.444% EAP, which closely matched response surface model predictions and ensured consistent raw material acquisition. For purification, the TCA method achieved 96.84% deproteinization efficiency, while subsequent AB-8 macroporous resin adsorption removed phenolic compounds, pigments, and other small molecules, resulting in a 76.04% polysaccharide retention rate and 83.80% purity. Structural analysis revealed that purified EAP is a neutral pyranose polysaccharide with a weight-average molecular weight (Mw) of 1.739 × 10^5^ g/mol, primarily composed of glucose, mannose, and galactose in a 0.470:0.199:0.081 molar ratio. In vitro digestion experiments demonstrated that EAP is a partially digestible polysaccharide with 3.30% cumulative digestibility: it remained stable in the oral stage, partially degraded in the gastric environment, and continuously degraded in the intestinal environment, indicating its potential to function as a sustained-release substrate in the gastrointestinal tract. This systematic investigation—from efficient extraction and standardized purification to structural identification and digestive characterization—clarifies the fundamental physicochemical properties and digestive behavior of EAP. These findings provide reliable data and a theoretical foundation for elucidating its metabolic mechanisms, exploring biological functions, and developing EAP-based functional food products with targeted gut health benefits.

## Figures and Tables

**Table 1:** Box–Behnken design with three levels based on single-factor optimization.

Std	Run	A: Ultrasonic Power (W)	B: Ultrasonic Temperature (°C)	C: Ultrasonic Time (min)	D: Liquid-to-Solid Ratio (mL/g)	Yield (%)
17	2	120	50	20	40	0.889
14	16	140	60	20	40	0.567
7	15	140	50	20	50	0.720
13	28	140	40	20	40	0.867
5	11	140	50	20	30	1.137
18	14	160	50	20	40	0.705
3	3	120	60	30	40	1.032
1	13	120	40	30	40	1.028
11	4	120	50	30	50	1.050
9	10	120	50	30	30	1.452
24	17	140	60	30	50	0.905
21	18	140	40	30	30	1.213
23	29	140	40	30	50	1.273
22	21	140	60	30	30	1.409
29	25	140	50	30	40	2.377
27	6	140	50	30	40	2.391
25	22	140	50	30	40	2.394
28	26	140	50	30	40	2.412
26	23	140	50	30	40	2.457
4	12	160	60	30	40	1.062
10	7	160	50	30	30	1.204
12	27	160	50	30	50	1.327
2	9	160	40	30	40	1.334
19	5	120	50	40	40	0.487
6	24	140	50	40	30	0.521
16	1	140	60	40	40	0.547
15	19	140	40	40	40	0.582
8	20	140	50	40	50	0.963
20	8	160	50	40	40	0.847

**Table 2:** Analysis of variance of the regression model (data as in the original manuscript).

Source	Sum of Squares	df	Mean Square	F-Value	*p*-Value	
Model	10.62	14	0.7585	215.63	<0.0001	significant
A—ultrasonic power	0.0220	1	0.0220	6.26	0.0254	
B—ultrasonic temperature ratio	0.0560	1	0.0560	15.93	0.0013	
C—ultrasonic time	0.0733	1	0.0733	20.84	0.0004	*
D—liquid-to-solid	0.0406	1	0.0406	11.54	0.0043	
AB	0.0230	1	0.0230	6.52	0.0229	
AC	0.0740	1	0.0740	21.03	0.0004	*
AD	0.0689	1	0.0689	19.59	0.0006	
BC	0.0176	1	0.0176	4.99	0.0423	
BD	0.0795	1	0.0795	22.61	0.0003	*
CD	0.1845	1	0.1845	52.44	<0.0001	**
A2	2.48	1	2.48	704.47	<0.0001	**
B2	3.11	1	3.11	885.16	<0.0001	**
C2	7.30	1	7.30	2074.19	<0.0001	**
D2	1.74	1	1.74	494.48	<0.0001	**
Residual	0.0492	14	0.0035			
Lack of fit	0.0454	10	0.0045	4.72	0.0741	not significant
Pure error	0.0038	4	0.0010			
Cor total	10.67	28				

** = extremely significant; * = significant.

**Table 3:** Comparison of different deproteinization methods.

Reagent	Concentration (mg/mL)	Absorbance Value	Protein Removal Rate (%)
Trichloroacetic acid	0.123	0.080	96.84
n-Butanol	0.637	0.380	83.70
Trichloroacetic acid + n-butanol	0.497	0.298	87.29

**Table 4:** Physicochemical properties of EAP before and after purification.

Component	Before Purification (%)	After Purification (%)
Total sugar	87.83 ± 0.12 ^a^	91.07 ± 0.06 ^b^**
Protein	39.93 ± 2.89 ^a^	2.37 ± 0.35 ^b^**
Reducing sugar	47.73 ± 4.57 ^a^	28.73 ± 1.03 ^b^*
Ash	12.62 ± 0.10 ^a^	5.04 ± 0.20 ^b^***

Note: All contents are expressed as percentages of the sample dry weight (%, *w*/*w*). Values are presented as means ± standard deviations (n = 3). Different superscript letters (^a^ and ^b^) indicate significant differences between before and after purification (*p* < 0.05). All four components showed statistically significant differences between the two groups. Asterisks indicate the significance level compared to before purification: * *p* < 0.05, ** *p* < 0.01, and *** *p* < 0.001.

**Table 5:** Monosaccharide composition of EAP.

Monosaccharide	Peak Area (nC·min)	Retention Time (min)	Molar Ratio	Content (μg/mg)
Fucose	0	5.109	0.000	0.000
Galactosamine	1.114	10.15	0.018	0.129
Rhamnose	0	10.75	0.000	0.000
Arabinose	0.377	11.667	0.022	0.106
Glucosamine	2.095	13.025	0.044	0.307
Galactose	0.807	15.15	0.081	0.475
Glucose	11.51	17.325	0.470	2.756
Xylose	1.263	20.467	0.091	0.443
Mannose	2.201	21.592	0.199	1.167
Fructose	0	25.075	0.000	0.000
Ribose	0	27.334	0.000	0.000
Galacturonic acid	0.139	42.892	0.022	0.137
Guluronic acid	0	43.625	0.000	0.000
Glucuronic acid	0.796	45.392	0.054	0.340
Mannuronic acid	0	47.792	0.000	0.000

Note: Content is expressed as μg monosaccharide per mg of lyophilized purified polysaccharide (dry weight, DW basis). The monosaccharide contents represent the measured values in the hydrolysate, while the total sugar content was determined by the phenol-sulfuric acid method. The calculation bases for these two measurements are different. nC·min = nanoCoulomb·minute (peak area unit for HPAEC-PAD detection).

**Table 6:** Molecular weight parameters of EAP.

Peak Number	TIME (min)	Mn (g/mol)	Mw (g/mol)	Mw/Mn	Mass Fraction (%)
peak 1	12.074–17.012	1.175 × 10^6^	1.417 × 10^6^	1.206	4.1
peak 2	17.012–19.241	3.929 × 10^5^	5.975 × 10^5^	1.521	5.1
peak 3	19.241–26.218	1.129 × 10^5^	1.739 × 10^5^	1.540	90.8

## Data Availability

The original contributions presented in this study are included in the article. Further inquiries can be directed to the corresponding author.

## Abbreviations

The following abbreviations are used in this manuscript:

ANOVA	Analysis of variance
BBD	Box–Behnken design
TCA	Trichloroacetic acid
DEAE-52	Diethylaminoethyl cellulose-52
DNS	3,5-Dinitrosalicylic acid
EAP	*Elaeagnus angustifolia* polysaccharide
FT-IR	Fourier-transform infrared spectroscopy
GPC	Gel permeation chromatography
HPLC	High-performance liquid chromatography
Mw	Weight-average molecular weight
PDI	Polydispersity index
RSM	Response surface methodology
SD	Standard deviation
SEM	Scanning electron microscopy
SGF	Simulated gastric fluid
SIF	Simulated intestinal fluid
SSF	Simulated salivary fluid
UV	Ultraviolet spectroscopy
